# Supplementary material for: From carbohydrates to fat: Trends in food intake among Swedish nutrition students from 2002 to 2017
Source: PLoS One. 2020 Jan 28;15(1):e0228200. doi: 10.1371/journal.pone.0228200 (PMC6986719; doi:10.1371/journal.pone.0228200)
Supplement: Attachment — Submitted filename: Response to reviewers PONE-D-19-24477.docx [file pone.0228200.s002.docx]

Response to reviewers PONE-D-19-24477

From carbohydrates to fat: Trends in food intake among Swedish nutrition students from 2002 to 2017

Maria Bergström, Andreas Håkansson, Anna Blücher and Håkan S. Andersson

We are grateful for constructive comments and suggestions for improvements regarding our manuscript. We believe that we have been able to meet all remarks from reviewers and will explain our actions below.

**Response to Reviewer #1.**

Comment 1: We agree with the reviewer that the manuscript benefits from an improved description of what data that was statistically evaluated. We have added one figure (as suggested by Reviewer 2) and changed the phrasing in the abstract to clarify that data from 2009-2017 was in focus for the statistical evaluation. We do however believe that it is important to present data from the beginning of the period (2002-2008) to allow comparisons of the long-time changes evident in our data to those of official sources, as presented in Figure 3 in the revised manuscript.

Comment 2: Revised manuscript is modified according to Reviewer´s suggestion.

Comment 3: Revised manuscript has been rephrased to reflect that the Northern Sweden Diet Database (NSDD), which is part of the Västerbotten intervention program (VIP) and the Northern Sweden MONICA project, are large ongoing independent cross-sectional health surveys. The data that we use was not part of any intervention program and line 77 has been changed.

Comment 4: Revised manuscript is modified according to Reviewer´s suggestion.

Comment 5: Revised manuscript is modified according to Reviewer´s suggestion.

Comment 6: It is our understanding that figure number should not be included in the figure; only in the figure legend.

Comment 7: Revised manuscript is modified to clarify that it is the average daily intake for each individual (mean value of two reported days) that is used in all calculations.

Comment 8: It is difficult to say to what extent the intake of male consumers with the same demographic characteristics would differ from the female ones. Unfortunately, the number of male participants was too low to allow a meaningful comparison between female and male consumers. This is a limitation of the study, and we have clarified in the introduction that only women were included. This is also evident in the new figure added to the manuscript.

Comment 9: Almost all excluded values were due to underreporting, similar to what has been seen in previous investigations and the revised manuscript has been modified to reflect this fact. A median value, as an alternative to Goldberg cut-off, is likely to skew the results as the number of participants eating below cut off by far exceeds the number eating beyond cut off.

Comment 10: Revised manuscript is changed according to Reviewer´s suggestion.

Comment 11: Standard energy conversion factors were used in the calculations and total energy was calculated as: protein x 17 kJ/g + fat x 37 kJ/g + carbohydrates 17 kJ/g + fiber 8 kJ/g + alcohol 29 kJ/g. This information has been included in the methodology section. Soluble and insoluble fiber were not identified; only total fiber.

Comment 12: Height and weight were self-reported by the participants. This has been included in the methodology section and discussion in the revised manuscript.

Comment 13: Revised manuscript is changed according to Reviewer´s suggestion.

Comment 14: Official statistics of food consumption in Sweden 1980-2016 was extracted from the statistical database available online from the Swedish Board of Agriculture as the mean intake of protein, fat, carbohydrate, fiber and alcohol expressed as kJ/day, which were transformed into E%. These data as well as E% values reported in the national surveys performed by the Swedish National Food Agency in 1988, 1997-1998 and 2010-2011 were used for comparison with the results in the present study. The manuscript has been changed to reflect this.

Comment 15: Revised manuscript is changed according to Reviewer´s suggestion.

Comment 16: Revised manuscript is changed according to Reviewer´s suggestion.

Comment 17: Micronutrients included in the study were selected based on the latest national (Swedish) food survey, reporting that their intake contrasted relative to the Nordic nutrition recommendations. This is clearly stated in the revised manuscript.

Comment 18: Page numbers have been corrected.

Comment 19: We have reformulated the text to clarify that the difference between 2002 and 2017 in vitamin D intake is not statistically significant (p=0.38), but a positive linear trend was observed over the time interval (p=0.01).

Comment 20: Revised manuscript is changed according to Reviewer´s suggestion.

Comment 21: Values in table 3 do not have a normal distribution and median values were used in the statistical evaluation (not mean values). Standard deviation has not been calculated for these values as the Wilcoxon rank sum test was used in statistical analysis.

Comment 22: The number of drop-outs from the course was 34% among women, which involved students who took part in the initial phases of the course, but who did not complete the food survey. We have rephrased the text in the methodology section and included a remark in the discussion to address this fact.

Comment 23: We agree with the reviewer that it would be most interesting to know more about the intake of whole grains in our study. We have used the standardized food categories used by the National Food Agency in Sweden, and these do not differentiate between whole grain products and products based on refined flour. We can however see in our data that intake of nuts and grains has a distinctive increase, which we also discuss. We have added a paragraph to discuss the limitation of food categories and also the need of improving food databases in this respect.

Comment 24: Revised manuscript is changed according to Reviewer´s suggestion.

**Response to reviewer #2**

1. The importance of food groups and variability among databases are discussed, and the work of Marconi, S., Durazzo, A., and Camilli, E. et.al is acknowledged.

2. A scheme of design of the study has been added to the revised manuscript.

3. A conclusion has been added to the revised manuscript as suggested by the reviewer.
